# All That Glitters Is Not Grit: Three Studies of Grit in University Students

**DOI:** 10.3389/fpsyg.2018.01539

**Published:** 2018-08-29

**Authors:** Chathurika S. Kannangara, Rosie E. Allen, Gill Waugh, Nurun Nahar, Samia Zahraa Noor Khan, Suzanne Rogerson, Jerome Carson

**Affiliations:** ^1^Psychology, Faculty of Professional Studies, University of Bolton, Bolton, United Kingdom; ^2^Education, Faculty of Professional Studies, University of Bolton, Bolton, United Kingdom; ^3^Institute of Management, University of Bolton, Bolton, United Kingdom

**Keywords:** retention, success factors, engagement, grit, university students, academic achievement, resilience, mixed-methods

## Abstract

The present research looked at the importance of the concept of grit in University students based on a mixed-method approach. Study 1 comprised 440 University students. All were given the Grit Scale, the Perceived Stress Scale, the short Warwick–Edinburgh Mental Well-being Scale, the Office of National Statistics Well-being items and the Self-Control Scale. Levels of grit were significantly higher in female students, older students and postgraduates. Grit correlated highest with self-control. Study 2 looked at 340 University students. In addition to measuring self-control, mental well-being and grit, measures of resilience and mindsets were also added. A construct validity test of the Grit Scale showed that high grit scorers had significantly higher levels of self-control and mental well-being, were more resilient and were more likely to have a more growth oriented mindset. Grit varies with age and is most closely associated with the concept of self-control. The third study was a qualitative investigation with 10 successful graduates. Semi-structured interviews were coded using thematic analysis. Three broad themes emerged. The first, Passion and Perseverance, included themes of having short and long terms goals, resilience, dedication, and endurance. The second, Self-Control, included time management, self-awareness, prioritizing tasks and knowing strengths and weaknesses. The third theme identified was Positive Mindsets. This included having a positive attitude toward learning, the importance of feedback and constructive criticism and that success is not materialistic. The qualitative research has helped “unpack” concepts from the grit research and may enable University tutors to guide students better. Though these studies were only conducted in one English University, they have been stepping stones in our quest to discover what are the most important factors in determining student academic success? The development and piloting of our new Uni-Stride Scale, is the next step in this process.

## Introduction

The concept of grit, originally articulated by Duckworth et al. (2007), has developed and expanded in parallel with the field of positive psychology. Indeed Seligman (2011) in his major textbook *“Flourish,”* devoted an entire chapter to the concept. Of course further research will either lead to the consolidation of the importance of grit, or will suggest there are other more important constructs, such as the longer established concept of resilience (Werner, 1996). In this paper, we began by reviewing the literature on grit, they then describe three studies they conducted on University students, two quantitative studies and one qualitative investigation. Finally, we mention the potential promise of a brief new measure of student resilience, Uni-stride, which we are currently researching. First, we looked at the concept of grit.

One of the pillars of Psychology has been research into intelligence, which has looked at both theoretical and applied aspects of the concept. Clinical psychologists have relied on various iterations of the intelligence scales originally developed by Wechsler (1955). This is now on its fifth version (Wechsler, 2010; Hubbard and Hare, 2015). Though called the WAIS IV (the first version was called the Wechsler–Bellevue Scale, but the Bellevue Hospital name was dropped from all subsequent versions). However, research began to show certain contradictions that revealed individuals of an equal or lesser IQ were consistently outperforming their “more intelligent” counterparts (Duckworth et al., 2007). Indeed, in many cases, individuals with a lower or average IQ were achieving higher qualifications, obtaining more influential job roles and receiving a higher income (Duckworth et al., 2007). Subsequently, there was a shift in research focus toward the importance of non-cognitive traits and factors in predicting and measuring achievement and success. Although the concepts such as perseverance, mindsets and goal driven behaviors which are identified to be non-cognitive traits were studied earlier in the field of education (Londoner, 1972; Levy and Dweck, 1998) a greater focus on the importance of these non-cognitive traits in this field was populated by Angela Duckworth, who was mainly concerned with the concept of grit. This can be divided into two sub-components; perseverance of effort and consistency of interest and the importance of fostering grit to enhance personal achievement and success. Duckworth et al. (2007, pp. 1087–1088) defines grit as the *“perseverance and passion for long-term goals”* and states that it involves *“working strenuously towards challenged, maintaining effort and interest over years despite failure, adversity, and plateaus in progress.”* Also, Duckworth stresses the importance of stamina in grit, and describes a “gritty” individual as somebody who treats their success and achievement as a marathon, rather than a sprint. Prior to Duckworth developing her research into non-cognitive predictors of academic success, educational research had focused on the more traditional measures of academic outcomes and a less focus into non-cognitive traits. Thus, the research of Duckworth generated a shift in research focus into predominantly considering non-cognitive traits, such as grit, that showed an individual’s character was much more influential.

Throughout the last decade of research into the construct of grit and its many applications, it has been recorded that there are certain demographic differences in grit. Findings have been published with regard to group differences in grit. Certain individuals are said to be more likely to develop grit and persevere in the face of adversity. Grit has been shown to correlate with gender (Flaming and Granato, 2017), with females scoring higher in grit than their male counterparts (Jaeger et al., 2010; Christensen and Knezek, 2014; Aswini and Amrita, 2017); and age (Cupitt and Golshan, 2015).

Grit has been posited as a highly influential construct linked to academic success and achievement. It has been found that grit is associated with academic productivity and engagement (Hodge et al., 2017); academic motivation (Eskreis-Winkler et al., 2014); academic achievement (Pate et al., 2017); perseverance in challenging tasks (Lucas et al., 2015); academic performance (Kelly et al., 2014); amount of hours studying (Cross, 2014); learning strategies (Weisskirch, 2016); task values and goal orientation (Muenks et al., 2016, 2017); the pursuit and attainment of postgraduate training (Palisoc et al., 2017), and the retention of students (Crede et al., 2016). Again there are also studies suggesting that grit is not a predictor of academic achievement and performance (Ivcevic and Brackett, 2014; Bazelais et al., 2016; Muenks et al., 2016; Palisoc et al., 2017).

As well as the construct of grit being associated with academic outcomes, some research has demonstrated an association between grit and certain personal outcomes. Grit has a significant relationship with higher life-course accomplishment (Abuhassàn and Bates, 2015); increased goal attainment (Sheldon et al., 2015); a diagnosis of a co-occurring psychiatric disorder (Griffin et al., 2016); employment status (Griffin et al., 2016); burn out (Halliday et al., 2016) and greater health care management skills (Sharkey et al., 2017). It is therefore worthwhile exploring the relationship that grit might have with student well-being and physical health.

Given the expansion of research into grit, it is important to measure the relationship that grit has with other related constructs such as well-being, self-control, resilience, mindsets, and perceived stress. To date, previous studies have indicated the strong association that grit has with certain psychological outcomes, reinforcing the notion that grit is strongly related to well-being. It has been shown that grit is positively correlated with happiness (Singh and Jha, 2008); satisfaction and a sense of belonging (Bowman et al., 2015); purpose commitment (Hill et al., 2014); psychological well-being (Goodman et al., 2017); value and self-efficacy (Muenks et al., 2016, 2017); self-esteem (Weisskirch, 2016); a growth mindset (Duckworth et al., 2007; Hochanadel and Finamore, 2015); pursuing engagement and pleasure in life (Von Culin et al., 2014); higher mental health (Sharkey et al., 2017); emotional stability during stressful or negative life events (Blalock et al., 2015); and a sense of meaning in life (Von Culin et al., 2014). Negative correlations between grit and certain psychological outcomes have also been established. Research has revealed a negative correlation between grit and perceived stress, arguing that “psychological resources, particularly grit, make students less prone to stress.” While there is a positive association between perceived academic failure and stress (Lee, 2017). This research suggests that focusing on stress management may reduce perceived academic failure, enhance grit and, in turn, improve academic success and personal achievement. Research evidence has also emerged that does not support an association between grit and certain psychological outcomes. Indeed, some research has found that grit was not a predictor of life satisfaction or depression (Vela et al., 2017). Thus, the relationship between grit and certain outcomes, again, has been shown to be uncertain. Future research should consider and address these inconsistencies in an attempt to establish a firm understanding and grounding knowledge of grit’s applications to certain success-related outcomes. It is identified that the mental well-being may influence not only factors determining our perceptions, thoughts, and behaviors, but also our physiological wellbeing (Huppert, 2009; Mullins et al., 2017). This study aims to include a mental well-being measure as a variable which will address the need of exploring the relationship between grit and psychological outcomes.

Additionally, previous research has indicated that grit is strongly correlated with self-control (Duckworth and Gross, 2014). Indeed, individuals with a high level of self-control may have the ability to regulate attention and resist temptation, but this does not necessarily mean they are persistent in attaining a particular goal in life. Individuals reported as being “gritty” may cease in the presence of temptation. Thus, the correlation between grit and self-control is strong, but not consistent as they engage and act differently (Duckworth and Gross, 2014). Furthermore, it would be worthwhile to understand the true relationship between self-control and grit in relation to academic success and personal achievement in students.

Similarly, little research has examined the relationship between grit and resilience as separate constructs. Duckworth herself suggests that resilience is a component of grit (Duckworth et al., 2007, Duckworth and Quinn, 2009), yet one study directly compared grit and resilience and found a negative correlation between the two constructs (Hardeman, 2016). Individuals who showed high levels of grit, showed low levels of resilience (Hardeman, 2016). These findings appear to be counter-intuitive, leaving future research with the need to explore these constructs more closely. Therefore, it is essential to truly examine the relationship between grit and resilience as two separate constructs that are seemingly related.

Perhaps not surprisingly in a time of major neuroscientific advance, recent research has also revealed an association between grit and personal achievement linked to the brain activation of a specific area of the brain. The neural substrate for grit has been identified as being located in the dorsomedial prefrontal cortex (DMPFC), the region also said to be responsible for concepts such as self-regulation, planning, goal-setting, and reflection of past experiences (Wang et al., 2017). However, this structural knowledge regarding the neural basis of grit should not discourage the idea that grit is a construct that can be fostered and built upon. Indeed, a growth mindset has been strongly associated with the presence of grit in individuals (Hochanadel and Finamore, 2015) and an academic environment that promotes growth is likely to foster gritty students who will learn to persist through challenges (Duckworth et al., 2007; Hochanadel and Finamore, 2015). The current study will explore ‘grit’; one’s passion and perseverance and its association to other factors such as self-control; the ability to control one’s emotions and desires in challenging or difficult situations, resilience; one’s ability to bounce back when faced with adversities, mental well-being; state of wellbeing in which one realizes his or her own potential, cope with normal life stressors and productivity, and growth mindsets; the capacity to believe that one’s most basic abilities can be developed through dedication and hard work.

### Present Studies

Clearly, the presence of “grit” within an individual is a hugely beneficial characteristic, as being a “gritty” individual has been associated with several factors relating to success and personal achievement. However, reservations have been expressed regarding the reliability of using grit as a significant measure or predictor of success. Research suggests that designing and orientating interventions that are focused on fostering grit will not have the desired effect on success (Crede et al., 2016). With the construct validity of grit in question, it is vital to determine whether grit is, in fact, a reliable measure of success and personal and academic achievement and how it relates to other constructs and factors relating to success.

The aim of this research is to address and explore the construct validity of grit as a significant and contributing factor toward certain outcomes, and to examine its usefulness in determining student success. Grit scores will provide crucial information about an individual that is distinct from the information gained from the measurement of other constructs. Therefore, the present research will aim to understand the incremental validity of grit. Study 1 will also examine any correlations with important factors relating to success and achievement such as self-control, resilience, psychological well-being, mindset and perceived stress. Study 2 will further explore the construct validity of grit by breaking up the participants into groups according to their total grit score. Namely, they will be grouped into a high scoring, average scoring or low scoring group and then the relationship between high and low grit scores and other variables can be examined. A third study is a qualitative study using a semi-structured interview approach with successful graduates from an English University. This aims to explore the relationship between, and to thoroughly understand, the qualities of exceptional students in relation to retention, learning strategies, perseverance, long-term goals and future success after graduation. Therefore, this research examines the meaningfulness and construct validity of grit in relation to other constructs. It also attempts to offer grit as a valuable construct that other universities should consider in measuring and predicting students’ success.

## Study 1

Study 1 aimed to distinguish any significant relationships between the construct of grit and other constructs such as self-control, well-being and perceived stress. By investigating the validity of grit as a meaningful construct that has positive and negative correlations with other constructs, this study will aim to validate grit as a useful construct. This study aims to explore the demographic predictors of grit by investigating the age and gender of the participants, and it was hypothesized that there would be an age-related trend shown in grit scores, with grittiness increasing with age. It was also hypothesized that gender would be a significant predictor of grit, with females scoring higher than males. With previous research in mind, it was hypothesized that grit would positively correlate with self-control and mental well-being, but that it would correlate negatively with perceived stress. It was also hypothesized that grit would positively correlate with life satisfaction, feelings of a worthwhile life and the degree of trust held in others.

### Method

#### Participants

A total of 440 foundation, undergraduate and postgraduate students from a University in the North-West of England were recruited. Participants’ gender, age, and current level of study were reported. The sample comprised 245 female participants and 170 males. Participants fell into the following age categories, 161 participants were between 18 and 21; 112 participants were between 22 and 26; 40 participants between the age of 27 and 30; 124 participants were 31 and above. The vast majority of participants were undergraduate students (357) with only 39 postgraduate student participants. Participants were recruited from different years of study. There were 18 students in their foundation year, 144 students in their first year; 103 students in their second year and 107 students in their final year.

#### Materials

Along with the following questionnaires, a brief outline of the task was provided, stating what would be expected of the participants, as well as a short explanation about what the study was investigating and the reasons for doing so. Participant information sheets outlined the participant’s ethical rights and identified that anonymity and confidentiality would be assured. A consent form was issued to each participant whereby they had to confirm their understanding of the study and what was required of them, and their agreement to take part in the study. The ethical rights of participants were also stated, such as the right “to withdraw at any stage up until the point that the questionnaire was handed back,” and that all participants were to be over the age of 18.

Each participant was required to complete a series of questionnaires including:

##### 12-item Grit (Duckworth et al., 2007)

The 12-item Grit Scale that was developed by Duckworth et al. (2007) was used for the purpose of this study. The scale was scored in the form of a Likert scale, asking students to respond to 12 statements, grading their answer from 1 to 5 depending on how they felt about each statement. For example, item 7 states “I often set a goal but later choose to pursue a different one” – with 1 being “not like me at all” and 5 being “very much like me.” The scale demonstrates high internal consistency at 0.85.

##### Perceived Stress Scale (PSS) (Cohen et al., 1983)

The Perceived Stress Scale requires participants to grade their responses to 10 statements in the form of a Likert scale. Statements include “I have confidence in handling personal problems” and “I am able to control irritations in my own life. The KMO coefficient of PSS was 0.82. A Cronbach’s alpha coefficient of 0.72 was reported for PSS. This confirmed the strong internal consistency and stability of the scale through repeated measures tests (0.93).

This measure has been widely used in surveys of student populations.

##### Mental Well-being Scale (*Short Warwick–Edinburgh Mental Well-being Scale*)

To measure participant’s mental well-being, the short Warwick–Edinburgh Mental Well-being Scale was used (Stewart-Brown et al., 2009). This is a 7-item scale that is scored in the form of a Likert Scale with 1 being “none of the time” and 5 being “all of the time.” Sample items are, “I’ve been feeling close to other people” and “I’ve been feeling optimistic about the future.” A Cronbach’s alpha score of 0.89 (student sample) and 0.93 (population sample) suggests some item redundancy in this scale. Test–retest reliability at 1 week was reported to be high (0.83). This scale is now one of the widest used measures of mental well-being in the world literature (Lloyd and Devine, 2012).

##### Personal Well-being (Office for National Statistics, 2011)

The measurement of participant’s personal well-being through a 4-item questionnaire that was adapted from the ONS Annual Population Survey required students to rate their response to four questions on a scale from 0 to 10 – with 0 being “not at all satisfied” and 10 being “completely satisfied.” Each question was focused around a particular concept: satisfaction, worth, happiness, and anxiety. For instance, the satisfaction question asked “Overall, how satisfied are you with your life nowadays?” The four items are considered to be independent of each other.

##### Self-Control Scale (SCS) (Tangney et al., 2004)

A 10-item self-scoring Self-Control Scale, adapted from Tangney et al. (2004) was used for this study. Again, the scale was scored in the form of a Likert scale with participants responding to 10 statements from “not at all like me” to “very much like me.” The scale included statements like “I get distracted easily” and “I’m good at resisting temptation.” Internal consistency estimates of reliability were high. Alphas for the Total Self-Control Scale were 0.89. Thus, the scale appears to have adequate internal reliability. Test–retest reliability was 0.89 for the Total SCS score and 0.87 for the SCS.

The Statistical Package for the Social Sciences (IBM SPSS Statistics Version 23) was used to analyze the results.

#### Procedure

Once the participants had read the information sheet and signed the consent form, they were asked to complete the study questionnaires. Participants were then thanked for their participation in the study and all documents were collected from each participant.

### Results

After the data was gathered, the results were collated, descriptive, and inferential statistics were conducted.

In order to accurately determine the effect of gender on grittiness, a randomized gender sample was used, where 150 randomly selected males and 150 randomly selected females were used in further analysis (see **Table 1**). *T*-test analysis found that females (*M* = 41.54, *SD* = 7.17) were significantly grittier than males (*M* = 39.26, *SD* = 6.74), *t*(281) = 2.748, *p* < 0.01. However, the effect size for this analysis (*d* = 0.33) was found to exceed Cohen’s (1988) convention for a small effect (*d* = 0.20).

**Table 1:** Demographic characteristics of the sample.

Demographic characteristic		Number of participants (*N*)	Percentage of sample (%)
Gender	Female	245	55.7
	Male	170	38.6
Age	18–21	161	36.8
	22–26	112	25.6
	27–30	40	9.2
	31 and above	124	28.4
Current level of study	Undergraduate	357	81.1
	Postgraduate	39	8.9

**Table 2** shows four significant findings between the total grit score of participants and their demographic characteristics. Participants who were aged 31 and above (*M* = 43.88, *SD* = 6.39) were significantly more likely to score high on grit than participants between the age of 18 and 21 (*M* = 38.63, *SD* = 6.89), *t*(268) = 6.376, *p* < 0.001. The effect size for this analysis (*d* = 0.78) was found to reach Cohen’s (1988) convention for a large effect (*d* = 0.80). Finally, analysis revealed that participants who were currently studying at a postgraduate level (*M* = 44.44, *SD* = 6.96) were significantly more likely to score higher on the grit score than participants who were currently studying at an undergraduate level (*M* = 40.10, *SD* = 6.98), *t*(375) = 3.457, *p* < 0.001. The effect size for this analysis (*d* = 0.62) was found to exceed Cohen’s (1988) convention for a medium effect (*d* = 0.50).

**Table 2:** Differences in grit scores by demographic variables.

Demographic characteristic		Total grit score (mean)	*SD*	Significance (2-tailed)	Effect size *(Cohen’s d)*
Gender	Female (*N* = 150)	41.54	7.17	0.006^∗^	0.33
	Male (*N* = 150)	39.26	6.74		
Age	18–21	38.63	6.89	<0.001^∗∗^	0.78
	31 and above	43.88	6.39		
Current level of study	Undergraduate	40.10	6.98	<0.001^∗∗^	0.62
	Postgraduate	44.44	6.96		

*^∗^Significant at <0.01, ^∗∗^significant at <0.001*.

Data analysis revealed several significant correlations between total grit scores and the other measures. Such that, **Table 3** displays the results of a 2-tailed Pearson’s correlation between the total grit score and the total scores of other measures that were distributed to participants (self-control, well-being, and perceived stress).

**Table 3:** Correlations between grit and other measures taken using the total grit score.

Variable	1	2	3	4
1. Grit	-			
2. Self-control	0.523^∗∗^	-		
3. Well-being	0.384^∗∗^	0.279^∗∗^	-	
4. Perceived stress	-0.105^∗^	-0.148^∗∗^	-0.308^∗∗^	–

*^∗^Significant at <0.05, ^∗∗^significant at <0.001*.

**Table 3** demonstrates significant correlations between total grit score and other measures taken. Such that, a correlation between the total grit score and the total self-control score showed significance from a 2-tailed Pearson’s correlation at *p* < 0.001. Also, a 2-tailed Pearson’s correlation showed a significant correlation between the total grit score and the total score on the mental well-being scale at *p* < 0.001. Finally, a correlation between the total grit score and the total score of perceived stress showed significance from a 2-tailed Pearson’s correlation at *p* = 0.033. Grit correlated highest with self-control.

### Discussion

While there are several previously published studies that indicate no gender differences in grit (Duckworth et al., 2007, Duckworth and Quinn, 2009; Ali and Rahaman, 2012), the current study found a significant relationship between grit and gender. That is, female students were more likely to score high on the Grit Scale, compared to male students. Correspondingly, a range of more recent literature concurs with the demographic differences demonstrated in this study, arguing that females are significantly more likely to be grittier than males (Jaeger et al., 2010; Christensen and Knezek, 2014). Possible reasons for this could be that females are arguably more competent in certain abilities that are related to an increased level of grit, such as multi-tasking (Kuptsova and Ivanova, 2016) and parental factors (Guerrero et al., 2016*).* Following the Duckworth et al. studies, several researchers have explored the correlation between grit and age and found that there is a positive correlation between grit and age (Cupitt and Golshan, 2015; Griffin et al., 2016). Consequently, the older an individual is, the higher their level of grit. Therefore the present study gains support from previous research as findings revealed that students above the age of 31 were significantly more likely to score high on the Grit Scale, compared to students between the ages of 18 and 21. This could be explained by the increased likelihood of older students having overcome more challenges and setbacks in life, as well as having generally more life experiences to draw upon. Students who were in postgraduate courses were more likely to self-report higher levels of grit as opposed to undergraduate students. Likewise, students in their final year of studying consistently reported higher levels of grit than students in their foundation or first year of studying. The positive correlation between grit score and current level and year of study can be explained by the positive correlation between grit and age – as age and current level and year of study also positively correlate. Nonetheless there is still research that contradicts this general finding, and suggests that there is no variation in grit scores depending on the gender and age of individuals (Duckworth et al., 2007, Duckworth and Quinn, 2009; Ali and Rahaman, 2012; Eskreis-Winkler et al., 2014).

Recent research has investigated the relationship between grit and self-control and revealed that they are, in fact, strongly correlated constructs (Duckworth and Gross, 2014; Oriol et al., 2017). An individual can display high levels of grit along with the presence of self-control and vice versa. Grit has been associated with excellent time-management and an increased level of self-awareness, which are core aspects of having self-control. However, it can also be argued whether this is a result of Jangle Fallacy which occurs when people use different terms to describe the same thing which is often compounded as a result of different vocabularies of various disciplines (Coleman and Cureton, 1954; Reeves and Venator, 2014). This may be an area for future investigation considering the closeness between the dimensions of self-control and grit. Some recent research evidence does not support an association between grit and certain aspects of well-being, essentially arguing that grit was not a significant predictor of depression and that grit does not directly increase life satisfaction (Vela et al., 2017; Jin and Kim, 2017). On the other hand, other studies have indicated a strong association between grit and several aspects of well-being, suggesting that individuals with a high level of grit have an increased likelihood of achieving higher levels of well-being (Muenks et al., 2016; Goodman et al., 2017; Sharkey et al., 2017). The current study demonstrates several findings that indicate a positive correlation between grit and well-being. Students who scored high on grit were significantly more likely to score high on the short scale of mental well-being (sWEMWBS). Furthermore, students who show grit in the pursuit of their long-term goals require certain aspects of mental well-being such as a sense that the world is coherent and an authentic connection with the self (Vainio and Daukantaitė, 2015).

## Study 2

Study 2 was conducted to further examine the construct of grit and its relationship with other known predictors of success. Much like study 1, self-control and well-being were measured and in addition, resilience and mindset were also measured to further validate the meaningfulness of grit as a construct to measure…Also, Study 2 will further explore the construct validity of grit by breaking up the participants into groups according to their total grit score. Namely, they will be grouped into a high scoring, average scoring or low scoring group and then the relationship between high and low grit scores and the other measures will be examined. It was hypothesized that grit would positively correlate with self-control, resilience, well-being, and mindsets as indicated by previous research. It was also hypothesized that participants who score highly on grit will also score highly on self-control, resilience, well-being and mindset.

### Method

#### Participants

A total of 340 foundation, undergraduate and postgraduate students from the University of Bolton were recruited. Participants’ gender, age and current level and year of study were reported. Some 184 of participants were female and 141 were male. Participants had a range of ages with 153 participants between 18 and 21, 83 participants were between 22 and 26, 28 participants between the age of 27 and 30, and 63 participants 31 and above. Participants were recruited from different years of study. Thirty students in their foundation year, 127 students in their first year, 65 students in their second year, and 78 students in their final year. Also, 24 participants were postgraduate students.

#### Materials

Much like Study 1, a participant information sheet was provided, and participants were required to provide their consent before participating in the study.

Each participant was required to complete a series of questionnaires. Many of the measures used in Study 2 were the same measures used in Study 1, including: the 12-item Grit Scale (Duckworth et al., 2007); the 10-item Self-Control Scale (Tangney et al., 2004); and the 7-item short Warwick–Edinburgh Mental Well-being Scale (Stewart-Brown et al., 2009). In addition the following measures were included in the Study 2.

##### Resilience (*Connor–Davidson Resilience Scale – CDRISC-10*)

The Connor–Davidson Resilience Scale (CD-RISC10) was utilized for this study, which is a 10-item resilience scale that is scored in the form of a Likert scale – from “not true at all” to “true nearly all the time.” For instance, statements included “I am not easily discouraged by failure” and “having to cope with stress can make me stronger.”

##### Mindsets (Diehl, 2008)

A 20-item mindset quiz was used, that involves identifying the extent to which participants agreed or disagreed with several statements. Participants responded with “strongly agree,” “agree,” “disagree,” or “strongly disagree.” Statements included “your intelligence is something very basic about you that you can’t change very much” and “the harder you work at something, the better you will be at it.” This mindset quiz was created by Diehl (2008) based on Dweck’s (2006) growth mindset work. This quiz can be accessed at http://www.classroom20.com/forum/topics/motivating-students-with.

#### Procedure

Once the participants had read the participant information sheet and signed the consent form, they were asked to complete a series of short questionnaires that required them to report their feelings in response to several statements in relation to five scales that were provided. The participants were thanked for their participation in the study and all documents were collected from each participant.

### Results

Data analysis found that students’ grit scores differed depending on certain demographic characteristics of the sample (**Table 4**). Again, the gender of participants had a significant impact on the total grit scores. The age of participants and their current year of study were also significant predictors of grit scores. That is, students aged 31 and above were more likely to have higher grit than students between the ages of 16 and 21 and students in postgraduate studies were more likely to have a higher grit score than students in their foundation year.

**Table 4:** Demographic characteristics of the sample.

Demographic characteristic		Number of participants (*N*)	Percentage of sample (%)
Gender	Female	184	54.1
	Male	141	41.5
Age	16–21	153	45
	22–26	83	24.4
	27–30	28	8.2
	31 and above	63	18.5
Year of study	Foundation	30	8.8
	First year	127	37.4
	Second year	65	19.1
	Third year	78	22.9
	Postgraduate	24	7.1

#### Correlation Results

Much like Study 1, in order to determine the effect of gender on grit scores accurately, a randomized gender sample was generated that took 100 random male participants and 100 random female participants. Analysis revealed that, much like Study 1, females were significantly grittier (40.93) than male participants (38.81), with significance (2-tailed) at *p* = 0.029 (**Table 5**).

**Table 5:** Differences in students’ grit scores by demographic variables.

Demographic characteristics		Total grit score (mean)	*SD*	Significance (2-tailed)	Effect size
Gender	Female	40.93	7.43	0.029^∗^	0.31
	Male	38.81	6.00		
Age	16–21	38.33	6.58	0.001^∗∗^	0.21
	31 and above	43.74	5.99		
Current year of study	Foundation year	39.38	4.28	0.043^∗^	0.83
	Postgraduate	43.39	7.55		

*^∗^Significant at 0.05, ^∗∗^Significant at <0.001*.

To test the construct validity of grit, the frequencies of the total grit score were identified and the cumulative percentiles for these scores were revealed. Three groups of grit scores were developed. A low grit score was identified as a score of between 1 and 35. An average grit score was identified as a score between 36 and 45 and a high grit score was identified as a score between 46 and 60. Analysis of these results revealed that 76 students were low on grit; 183 students had an average score of grit; while 73 students were high on grit. Of the 340 students who participated in this study, eight grit scores could not be analyzed as the data were incomplete. Analysis revealed that there were four significant findings when using the grit threshold as the grouping variable, between high grit scores and low grit scores and other variables known to predict success (**Table 6**). However, according to reported effect sizes, there is a large effect on grit by current year of study where as a small effect on grit by age and gender. Unlike Study 1, effect size is reported to be small on age for Study 2 whereas year of study demonstrates a similar finding to study 1.

**Table 6:** Relationships between grit scores and the other dependent variables – using the grit threshold.

Variable	Grit threshold	*N*	Mean	*SD*	Significance (2-tailed)	Effect size
Self-control	Low grit	74	26.73	7.445	<0.001^∗∗^	0.65
	High grit	72	38.10	5.647		
Resilience	Low grit	68	22.75	8.042	<0.001^∗∗^	0.52
	High grit	68	31.47	6.445		
Mental Well-being	Low grit	73	20.66	7.146	<0.001^∗∗^	0.53
	High grit	71	28.08	4.471		
Mindset	Low grit	65	35.91	6.368	<0.001^∗∗^	0.38
	High grit	67	41.49	7.117		

*^∗∗^Significant at the 0.001 level (2-tailed)*.

#### Predictive Results

Findings demonstrate that students scoring high or grit had better self-control scores (38.10 vs. 26.73), and better mental well-being (28.08 vs. 20.66). Study 2 added measures of resilience and mindsets. Students high in grit scored significantly higher on resilience (31.47 vs. 22.75) and on mindsets (41.49 vs. 35.91). Effect sizes indicate that self-control, resilience, and mental well-being are having a large effect where mindsets are demonstrating a medium effect.

To test whether the grit is dependent on the variables resilience, mental well-being, mindsets, self-control, and age group, a multiple regression analysis was performed. Tests for multicollinearity indicated that a very low level of multicollinearity was present (VIF = 1.771 for resilience, 1.744 for mental well-being, 1.126 for mind-sets, and 1.132 for self-control).

As shown in **Table 7**, the score on the Grit Scale was significantly predicted by the scores on the resilience, mindsets, mental well-being and self-control scales. The multiple regression model with all four predictors produced *R*^2^ = 0.494, *F*(5, 255) = 48.74, *p* < 0.001. As can be seen in **Table 7**, the Resilience, Self-Control scales at *p* < 0.001 level, Mindsets at *p* < 0.01 level and mental well-being at 0.05 level significant positive regression weights, indicating students with higher scores on these scales were expected to have higher grit scores, after controlling for the other variables in the model. The regression model formula predicting the grit score is as follows: 12.149 (constant) + resilience (0.204) + mental well-being (0.154) + mindsets score (0.142) + self-control (0.394) + age group (0.588). The standardized coefficients revealed that their combinations explained 49.4% of the variance in the Grit Scale.

**Table 7:** Predictors of grit according to multiple linear regression analysis.

Variable	Unstandardized coefficient		Standardized coefficient			95% confidence interval
	*B*	*SE*	*B*	*t*	*p*	
Constant	12.232	2.083	–	5.874	<0.001	[8.132, 16.333]
Resilience	0.218	0.056	0.227	3.858	<0.001	[0.107, 0.329]
Mental well-being	0.144	0.066	0.127	2.183	<0.05	[0.014, 0.275]
Mindsets	0.146	0.049	0.140	2.985	<0.01	[0.050, 0.242]
Self-control	0.420	0.042	0.466	9.906	<0.001	[0.336, 0.503]
Age	0.588	0.287	0.098	2.049	<0.05	[0.023,1.153]

### Discussion

Much like Study 1, the total grit score of participants was significantly different depending on the gender of the participant. Similarly to Study 1 and also seen in the wider literature, there was an age trend in grit scores in Study 2 (Duckworth et al., 2007, Duckworth and Quinn, 2009; Cupitt and Golshan, 2015; Griffin et al., 2016). Indeed, students aged 31 and above were significantly more likely to report higher levels of grit than students between the ages of 16 and 21. Again, the positive correlation between grit and age further explains the positive correlation that was revealed between grit and the current year and level of study.

In the present study, resilience and mindsets were also measured along with grit, self-control, and mental well-being. Previous research into the relationship between grit and resilience is limited in the sense that they do not consider the two constructs as separate measures. However, research that does consider grit and resilience as separate measures found a negative correlation between the two constructs (Hardeman, 2016). Such that, an individual who showed high levels of grit, showed low levels of resilience (Hardeman, 2016). Explanations for these results are offered, suggesting that “grit is most often used as a predictor of achievement whereas resilience as a construct is much less goal oriented. What seems to be much more central to resilience are qualities such as adaptability and flexibility” (p. 55). In other words, grit is related to resilience, but an individual can be resilient while that individual is not necessarily gritty. The current study justifies the need to explore grit and resilience as two separate constructs that are strongly correlated as findings revealed that students who score high on grit are significantly more resilient than students who score low on grit. These findings contradict previous research, but could be explained by research that suggests that resilience is a component of grit (Duckworth et al., 2007, Duckworth and Quinn, 2009) and advocates the strong interrelated nature between the two constructs.

The positive relationship between grit and mindset that has been identified in already published literature is seen in the findings from the present study (Duckworth et al., 2007; Hochanadel and Finamore, 2015). Furthermore, students who scored high on the Grit Scale also scored high on the mindsets scale, suggesting that gritty students were significantly more likely to possess a growth mindset. Possible explanations for these findings include that the presence of grit is somewhat a natural practice for a student with a growth mindset. For instance, a student who believes that hard work will improve their skills and abilities is likely to be more determined and motivated to exert the effort to develop these abilities.

In contrary to some of the literature (Hardeman, 2016) suggesting significant independence between variables such as grit and resilience, the multiple regression results indicates that all four variables, i.e., resilience, mental well-being, mindsets, and self-control positively contribute toward ones grit score. Regression findings further confirms early work on grit conducted by Duckworth et al. (2007) and Duckworth and Quinn (2009).

## Study 3

In order to facilitate a qualitative perspective on grit’s relationship with success and academic outcomes, the third study was conducted. This study aimed to provide rich information about successful and gritty graduates from this University, which would allow generic themes to be identified. By applying principles of thematic analysis, this study aimed to draw conclusions surrounding the unique and influential traits that are present in successful graduate students.

### Method

#### Design

A solely qualitative methodology was adopted, gathering rich information about the unique qualities that are present in successful graduate students. Following the retrieval of information through semi-structured interviews, thematic analysis was carried out on the data.

#### Participants

Through a snow-ball sampling technique, a total of 10 successful graduates from the University of Bolton were recruited. Only students who graduated with a first-class honors degree and those who scored high on Grit Scale (score above 4, highest score on Grit Scale is 5) were considered for this study. Participants were both male (30%) and female (70%). The rationale behind this is due to the sole focus on gritty successful graduates and their qualities. In addition to graduating with a first-class degree from the University, many had extra academic awards.

#### Instruments and Procedure

An initial screening was conducted to identify students graduated with first classes followed by 12-item Grit Scale was presented to identified participants to confirm whether they are also high on grit. There were 10 questions that acted as a baseline for gathering information; however, when required, prompts and follow up questions were provided. The verbal consent of each participant was required. Ten open-ended questions were presented to each participant: focusing on personality characteristics, challenges they had faced, strategies to overcome these challenges, goals, and their opinions and perception of success and their attitude toward learning. For instance, participants were asked “When you started your course, did you aim to achieve a first-class degree?” They were prompted to think about certain obstacles they overcame in the pursuit of their degree by asking “What challenges have you faced during your time studying for your degree and what were your strategies to overcome these challenges?” The interviews were recorded and then transcribed with the permission of each participant. The anonymity of the participants was ensured as no personal or identifying information was withheld from recordings or disclosed in interview transcriptions. Recordings were destroyed after the interview had been transcribed.

Interviews were digitally recorded and transcribed verbatim. The qualitative data was analyzed using Braun and Clarke’s (2006) approach for applying thematic analysis in psychology. In order to ascertain and increase inter-coder reliability and the reliability of the results, six transcripts were independently coded by two of the paper’s authors to develop a coding framework and code book. Any subsequent additional themes were discussed during independent analysis. There was a 90 % agreement between the two coders for the theme grit/passion and perseverance and self-control, there was 80% agreement for growth mind-sets. However, there were other main themes that coders have independently identified such as ‘positive experiences’ and ‘hardships’ below the 50% agreement hence those themes were discussed between the independent coders, followed by and the sub-themes have been identified. When a lower level of agreement (50%) was identified between the coders after independent coding process, themes were discussed and a relevant and a most suitable theme was agreed. If there was a higher level of disagreement, those codes were removed from the analysis.

### Results and Discussion

The interview transcripts were examined to identify recurring patterns or themes, which were later identified as (a) passion and perseverance; (b) self-control; and (c) positive mindsets. These themes were made up of several sub-themes that appeared throughout the data, which have been highlighted in **Table 8**.

**Table 8:** Themes and sub-themes with example quotes from original text.

Themes (*N*)	Sub-themes (*N*)	Example quote
Passion and perseverance		
(*N* = 10)	Short and long-term goals identified (*N* = 10)	“Short-term I want to get onto a graduate scheme, I want to get into management, I need to lead people and I need to be the person in charge for a while…But to do that, I will need a Masters, but I don’t see the point in doing my Masters straight away without getting that experience of being a manger first.” (pp. 9, 81–86).
	Resilience – overcoming obstacles (*N* = 10)	“Being older, and a single parent with two kids, my mum told me I was thick every day through high school. She said get married, have kids, you don’t need a career. I mean my ex-husband was like what do you want to go to Uni for, you know you’ll get in loads of debt?” (pp. 8, 9–11).
	Motivation and dedication (*N* = 10)	“I always try and I don’t give up and I always push that little bit further.” (pp. 1, 19–20).
	Endurance (*N* = 6)	“So for me to go to university was more at the time to prove to people that I could do it, you know I may have disappointed people in the past but I wasn’t going to disappoint people now.” (pp. 3, 155–158).
Self-control		
(*N* = 10)	Time-management (*N* = 10)	“I’ll dedicate all my days in the week, 9 till 3.30 and I’ll go to the library and do what I’ve got to do. It was very rare that I had to dip into my weekends.” (pp. 4, 63–65).
	Self-awareness (*N* = 6)	“I think if I’d been 18 or 19, well I wasn’t ready. That’s why I didn’t do it, there was no way I could have got something big like this then.” (pp. 480–481).
	Prioritizing tasks (*N* = 7)	“That’s when I thought …well this one’s got to be done earlier, this one’s got to be done then and then I worked out which days of the week I was doing what work.” (pp. 8, 85–86).
	Aware of strengths and weaknesses (*N* = 8)	“I work a lot better under stress so that’s why I like exams so much. Although I think exams are not a good way of measuring intelligence, I’ve become very good at doing them.” (pp. 2, 85–87).
Growth mindsets		
(*N* = 10)	Positive attitude toward learning (*N* = 9)	“There is no point worrying about it the day before because while you’re worrying you could be revising, and not worrying about it during because you’re there so just get on with it, and there’s no point worrying about it afterwards because it’s over.” (pp. 2, 93–96).
	Importance of feedback and constructive criticism (*N* = 5)	“When I got a low mark I went for feedback, definitely, it’s important to know where you’re going wrong.” (pp. 4, 42–43).
	Success is not materialistic (*N* = 7)	“I wouldn’t say it’s a financial thing success. But I think if you end up getting into something that you enjoy doing and you find yourself in a position that you’re happy with and you enjoy, and you comfortable, then yes I would say you would be a successful person.” (pp. 9, 69–71).

*N, number of participants who mentioned each theme*.

#### Passion and Perseverance

According to Duckworth (2017), passion and perseverance are integral factors of grit. She argues that if an individual is pursuing a long-term goal that is aligned with their personal interests and they have a passion for the subject, that individual will persist to overcome challenges and achieve that goal (Duckworth, 2017). In light of this, the data analysis of third study revealed that successful students showed a lot of resilience in their time studying at University. Instead of buckling under pressure, or letting stressful life events consume their studies, they overcame these challenges and persisted in the face of adversity. Indeed, a few students faced academic challenges, with two of the students facing severe language barriers at the beginning of their degree courses. Two of the students did not speak good English when they started their University course and had to take English classes at the same time as studying for their full-time degree. This coincides with what Graham and Hanson (2013) stated. That is, by responding to challenges quickly, adaptively and effectively, gritty people will persevere through the set-backs as a result of their passion, perseverance, flexibility, and positively (Graham and Hanson, 2013). When discussing their recollection of their first day at university, one of these students stated that:

“*When I came to University I only really knew how to say “hello” and “goodbye”, very, very minimal, basic English. So I’ve done a couple of courses in English*” (pp. 5, 24–26).

So, not only did these students achieve first-class honors degrees, they also learned the English language in the process, which represents a huge level of determination and grit, for these individuals to persevere with their studies in addition to the language barriers they faced.

Along with language barriers, students faced personal challenges that included overcoming difficulties with employment, finances, relationships, family, childcare, bereavement, health, and adjustment to University life. Consequently, the majority of students, at some point during their studies, encountered challenges such as these. Childcare or health problems appeared to be a common pattern among the majority of successful students. When discussing obstacles they had overcome in their journey to achieve a degree, one student revealed that:

“*Having two children and I worked…because obviously we have to pay for a mortgage…. I lost two people while I was studying, who were very close so that was hard. Erm, but I also found that doing University stuff actually took my mind off it as well, in a way.*” (pp. 1, 35–39).

Interestingly, using University work, assignments and revision as a means of distraction from obstacles they faced, seemed to be commonly used by students. This coincides with what Duckworth coined as “passion,” as students use their studies as a distraction from their personal problems as their study tasks are aligned with their personal interests and make bouncing back from adversity an easier task (Graham and Hanson, 2013; Duckworth, 2017). Health issues were apparent for four of the 10 students, with one declaring that:

“*I have had four surgeries in the three years at University, been in hospital a lot and dealt with it. Just didn’t have the time to think about the illness.*” (pp. 5, 40–41).

Overcoming physical and mental health difficulties through adopting a positive attitude and focusing on the long-term goal was beneficial and helpful to some of the students. Challenges surrounding personal relationships surfaced a few times, with one student revealing that:

“*I had a break-up with the guy I was seeing – a raging alcoholic so that all went very badly wrong, so I wasn’t in the best place.*”(pp. 8, 25–27).

With this in mind, all students faced challenges in their studies that they overcame in order to achieve their degree and become successful graduates. The students’ academic resilience in persisting in the face of adversity suggests a strong personality that provides the determination to strive for their goals no matter what comes their way. The recurring theme among all students was their ability to manage their time effectively, ultimately succeeding in achieving the long-term goals they set themselves. Indeed, nine of the 10 students clearly identified their short and long-term goals. This gains further support from previous research that indicates gritty people are more likely to have their long-term goals and future plans identified (Muenks et al., 2016; Sheldon et al., 2015; Duckworth, 2017). One student who directly expresses this stated:

“*I’ve got a long-term goal and a long-term vision in my mind… I have got one and I am moving towards that.*” (pp. 4, 136–139).

As well as the students’ being goal driven, it was clearly evident that their motivational factors appeared to be similar throughout. Such that, seven of the students reported that they faced negative feedback or criticism from others about their abilities to accomplish their goals. The negative attitude of another person toward their studies showed students endurance to overcome the negative experiences and to continue in their goal pursuit. This resonates with previous research that illustrates the importance of self-efficacy and self-esteem in individuals with high levels of grit (Muenks et al., 2016; Weisskirch, 2016). Also, gritty people were found to have an increased sense of emotional stability during stressful or negative life events (Blalock et al., 2015*)* which relates to successful graduates overcoming emotional challenges in response to others doubting their ability to succeed. Furthermore, one student revealed that:

“*If my mum and ex-husband hadn’t been so down on me doing it, I might not have felt fired up enough to do it…”* (pp. 8, 132–133).

#### Self-Control

From the data analysis it was evident that the vast majority of successful graduates had excellent time-management and had a strong ability to dedicate specific times to University tasks (HOW MANY?). Instead of leaving assignments to the last minute, or attempting to complete essays the day before submission, students planned their time and set specific times when they would sit down to study. Seligman (2011) suggests there is a relationship between grit and self-control in the sense that gritty people are more likely to be self-disciplined and organize their time so that they can achieve their goals, expressing a high degree of self-control. For instance, one student divulged:

“*I always set deadlines, personal deadlines…always be ready at least 4 days before the actual deadline so I was left time of 3 or 4 days to double check, to proof read…*”(pp. 5, 46–48).

As well as the successful graduates utilizing their time-management abilities as a means for overcoming challenges; their ability to prioritize their tasks is apparent – as shown in **Table 8**.

A high degree of self-awareness and reflection is indicated through the analysis. Being aware of their current situation and drawing on past experience, the students were able to use this awareness as an advantage and incorporate it into their studies. This coincides with an earlier model that was proposed by Kanfer (1970) that shows how individuals with a high degree of self-control have the ability to learn reinforcement and control over their behavior. Through self-monitoring and self-evaluation, they are able to identify problematic behaviors and compare the outcomes of this behavior with their desired outcomes (Kanfer, 1970; Eysenck and Eysenck, 1981). This further relates to how gritty students tend to have an enhanced level of self-control as students’ awareness of their past experiences allows them to understand their strengths in a way that they can apply to different situations effectively (Lopez et al., 2015). For instance, one student reported that,

“*I can’t work in the evening, I get too tired.”* (pp. 3, 31–32).

By having an understanding of themselves and incorporating this awareness into their University life, they were able to successfully complete assignments and exams to a high standard. An aspect of self-awareness involves being aware of your own strengths and weaknesses and using them to your advantage. This, too, was evident from the data analysis. Half of the students identified an awareness of their strengths and weaknesses and how their understanding of them has benefited their success as a student. Again, this is reinforced by the notion that gritty individuals have a better knowledge and understanding about how they can evaluate their strengths and weaknesses, and further apply them in new ways to increase their likelihood of success in the future (Grenville-Cleave, 2012). Indeed, one student divulged:

*“I work a lot better under pressure…I work a lot better under stress so that’s why I like exams so much. Although I think exams are not a good way of measuring intelligence, I’ve become very good at doing them.”* (pp. 2, 79–87).

#### Growth Mindsets

The students’ attitude toward learning, and life in general was quite positive. Instead of focusing on the negatives, or stressing about the challenges they have faced, they persevere and adopt a positive outlook. These findings relate to the concept of positivity and the possession of a positive outlook on life and how this can foster a more suitable candidate for success (Fredrickson, 2009). Subsequently, see **Table 8** for an example. Additionally, when discussing strategies to overcome challenges that are faced when studying at university, one student explained that:

“*I don’t like the word expert – because I don’t think you will ever fully be one. I think you always have your weaknesses and I think if you’re in a job that allows you to see those weaknesses then that’s going to help you develop professionally and personally. So, I think the best thing about overcoming challenges is that you see it in your own reflection process, and then you’re like oh yeah I couldn’t do that a year ago but now I can.*” (pp. 10, 58–62).

With the majority of successful graduates sharing a positive attitude to learning, their ideas about what success is, were also similar. Analysis revealed that over half of the respondents did not believe that success was materialistic. Rather, they stressed the importance of happiness, independence, comfort and a stress-free life. Martin Seligman stressed the importance of learned optimism and how people can change their mind and ways of thinking in order to facilitate a better attitude toward life, with a more positive approach (Seligman, 2011). Successful students seemed to adopt this idea of learned optimism where they believed happiness and independence were crucial. For instance, one student communicated that:

“*There is a financial element to success, but it’s not that important to me, quality of life is important;*” (pp. 4, 112–113).

The importance of receiving feedback on assignments and the crucial element of constructive criticism was stressed by the majority of students. Using the opportunity to understand where they went wrong so students could improve next time, and so they could expand on their skills and abilities was deemed important. This directly represents the theory proposed by Dweck (2012). Subsequently, students seemed to possess a growth mindset, where they advocated the need for improvement and recognized the importance of development and growth in their abilities (Dweck, 2012). Indeed, one student commented that:

“*When I got a low mark I went for feedback, definitely, it’s important to know where you’re going wrong*.” (pp. 4, 42–43).

Even when students received high marks, they still believed feedback sessions were important. For instance, one student revealed that:

“*I used to always go for feedback on things, so even if I had got a first, a good grade, I’d still go and be like what else can I do? I don’t want to be complacent. How else can I push to get from the 70’s to the 80’s?”* (pp. 8, 137–139).

Thus, rather than focusing on the negative aspects of assignments and disregarding them once they were submitted, successful graduates appreciate the importance of feedback on their work and used this opportunity to further build on their work. Again, this is reflective of Dweck’s concept of a growth mindset – as successful students were eager to build on their existing skills in order to express personal development and growth (Dweck, 2012).

It is also important to note that successful students noticed the responsibility they held over their own success. While considering the support that other people and services have offered them, ultimately their success is down to their own actions. Indeed, one student represents this idea by stating:

“*Yeah I think to a point you can say it’s the individual who is to blame for the success and the achievement, because they are the ones who have accumulated it all, you put it all together, but it’s definitely not all me*.” (pp. 2, 134–136).

While some of the graduates were not quick to mention themselves in their own success and mentioned peers, tutors, family and friends as influencers of their success, the majority of students noticed that they played a huge role in their success as a student. For instance, one graduate expressed this directly when stating that:

“*I’m the one who put the work in. I know X [referred to one of the lecturers] encouraged me to put some work in and I strived to be like my brother, but at the end of the day I’m the one who is writing the essays*…”(pp. 9, 76–78).

Ultimately, successful graduates from this University showed passion and persevered toward achieving their degree despite setbacks. They demonstrated high levels of self-control and they embraced a growth mindset. These principle findings and core characteristics molded the graduates into the perfect candidates for success, as the vast majority of the successful students shared similar challenges, strategies, awareness, and perspectives throughout their studies at University. Furthermore, high achieving students showed the following attributes, an increased level of determination and grit; overcame personal and academic challenges; strongly identified their long-term goals and future plans; were trying to prove a point to someone else; possessed excellent time management and prioritizing abilities; expressed a high degree of self-awareness in their own strengths and weaknesses; occupied a positive attitude toward learning and a growth mindset; revealed the importance of happiness, independence, comfort and a stress free life; stressed the importance of receiving feedback and constructive criticism; and noticed the responsibility they held over their own success.

## General Discussion

Across all three studies, grittier students were more likely to demonstrate an increased level of self-control, mental well-being, life satisfaction, feelings of worth, resilience and growth mindset. Gritty students were more likely to illustrate a decreased level of perceived stress (Study 1). In addition, mature students were more likely to self-report higher levels of grit (Studies 1 and 2). High achieving, successful students were consistently more likely to have overcome personal challenges and obstacles and persevere despite facing obstacles and barriers (Studies 2 and 3). These students were able to strongly identify their long-term goals and strived toward the achievement of these goals with the intention to prove a point (Study 3). Excellent time-management and prioritizing skills were evident in successful students, along with their ability to express self-awareness (Study 3). These high-achieving students held a positive attitude toward learning and life in general and stressed the importance of feedback and constructive criticism in their personal development and growth (Study 3). Finally, these students did not believe success was materialistic. They stressed the importance of happiness, independence, comfort and a stress free life while recognizing the responsibility they held over their own success (Study 3). These principal findings demonstrate the core characteristics of high-achieving, successful students and illustrate what traits and characteristics are fundamental to academic success.

Measuring and monitoring certain traits and characteristics of students is evidently a worthwhile task. The current study reinforces the usefulness of measuring constructs such as grit and mental well-being, and reinstates the strong relationship they have with student success and academic performance. The knowledge that arises out of measuring students’ grit, resilience, mental well-being, mindsets, self-control, and other factors that have shown to be associated with academic success, provides institutions and organizations with valuable information about their student population. Furthermore, this provides Universities and other institutions the opportunity to monitor and regulate these hugely influential traits, ultimately enhancing their students overall mental well-being, which in turn, increases the likelihood of success and academic and personal achievement. With this in mind, the need for an academic resilience scale that can be used in any university or institution is very apparent. Indeed, the development of a concise scale that incorporates and measures all of these hugely influential factors is essential.

Previous research by author 1 that focused on languishing and thriving dyslexics included the development of a framework for dyslexia to work within positive psychology (Kannangara, 2015). This framework demonstrates the core characteristics of a languishing student, who focuses on weaknesses, has low grit and self-control, possesses a fixed mindset and has learned helplessness, and a thriving student who focuses on strengths, has an increased level of grit and self-control, possesses a growth mindset and has learned optimism. Furthermore, a thriving student has greater determination toward goals and persists in working toward these goals despite setbacks, embracing challenges instead of giving up. A thriving student recognizes the responsibility they hold over their own choices and actions and focuses on their strengths by applying them in different ways. Clearly, concepts of this framework can be applied to the academic resilience of students in that the framework reflects the principal findings of the current study. Indeed, it highlights the interrelated nature of these constructs, reinforcing the finding that they strongly correlate with each other and each have a significant role to play in student success.

**FIGURE 1:**
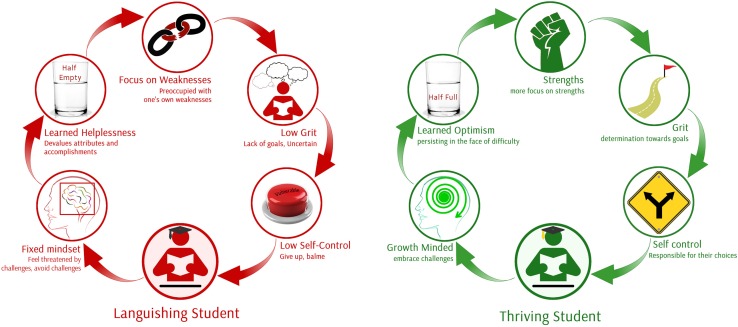
From languishing to thriving students. Model and the figure developed by Kannangara (2015).

## Limitations and Future Research

Although the current study generated several significant findings in relation to the meaningfulness of grit and its strong correlations with other constructs related to student success, the studies are not without their limitations. Firstly the current study only took part in one institution, a University in the North-West of England, which may also limit generalization. Thus, in order to truly represent the population and generate more reliable findings, future research should recruit students from different institutions, with a diverse sample of cultures, ages, genders, and backgrounds. This would yield a more conclusive and accurate relationship between grit, demographic differences, and success related factors, therefore providing crucial information about the construct of grit and which groups of the population should focus on their development of grit. Second, two of the studies used self-report measures of grit, self-control, mental well-being, resilience, mindset, and perceived stress, with all the objections that accrue from self-report, such as social desirability effects. It is a widely known fact that within psychological and educational research, self-report methodology dominates the study of student motivation. A review conducted by Fulmer and Frijters (2009) reports that the scope of motivation research can be expanded by incorporating a wider range of methodologies and measurement tools such as phenomenological, neuropsychological/physiological, and behavioral and related tools. As suggested by the review concerning motivation studies, the current study also can be expanded by integration or combination of these approaches and a preliminary functional framework for the development of novel, multidimensional approaches in order to study concepts such as grit, resilience, and self-control. Lastly, there are of course problems in drawing conclusions from cross-sectional studies only. More longitudinal studies are needed in this field.

However, the third qualitative study revealed some of the factors that may underlie grit in University students and merit further investigation. While all that glitters is not grit, the current studies suggest that Duckworth’s research requires additional validation. We are currently working on the development of a short 12 item measure, the Bolton Uni-Stride Scale (BUSS), to tap into many of the attributes identified in these studies to try and determine what factors lead to academic success at University.

## Ethics Statement

Approval for three studies were given by the University of Bolton Research Ethics Committee in 2016, United Kingdom.

## Author Contributions

CK, JC, and GW were involved in the conception and design of the studies. CK and RA organized the database of the three studies. NN, SK, and SR collected the data for Study 1, Study 2, and Study 3. CK, RA, and JC were involved in performing statistical analysis and thematic analysis. RA and CK wrote the first draft of the manuscript and all authors contributed to manuscript revision, read and approved the submitted version.

## Conflict of Interest Statement

The authors declare that the research was conducted in the absence of any commercial or financial relationships that could be construed as a potential conflict of interest.
